# Biallelic Heterozygous Mutations in Crumbs Homolog-1 Gene Associated With Macular Retinoschisis and Angle-Closure Glaucoma: A Case Report and Literature Review

**DOI:** 10.3389/fopht.2022.902898

**Published:** 2022-06-03

**Authors:** Jia-Xing Sun, Hong-Xiang Yan, Dan Hu, Jian Zhou, Yu-Sheng Wang, Jing Wu, Xiao-Jin Song, Xu Hou

**Affiliations:** Department of Ophthalmology, Eye Institute of Chinese PLA, Xijing Hospital, Fourth Military Medical University, Xi’an, China

**Keywords:** Crumbs homolog-1 (*CRB1*), macular retinoschisis, Coats-like vasculopathy, angle-closure glaucoma, mutation

## Abstract

**Background:**

Mutations in the Crumbs homolog-1 (*CRB1*) gene are associated with a variety of retinal degenerations including Leber congenital amaurosis (LCA) and retinitis pigmentosa (RP). It is also important to highlight atypical features to make proper diagnosis and treatment.

**Case Presentation:**

We present the case of a 7-year-old girl with biallelic heterozygous *CRB1* mutations. The clinical features include macular retinoschisis, Coats-like vasculopathy, short axial length, and angle-closure glaucoma (ACG). We also briefly review the current opinion on *CRB1* mutation-related diseases.

**Conclusion:**

*CRB1* mutations could result in a combined manifestation in anterior and posterior segments. This case emphasizes the importance of genetic diagnosis for those young patients with complicated rare clinical features to call for a specific treatment and follow-up plan. It also highlights the crucial role of *CRB1* in eyeball development.

## Introduction

The Crumbs homolog-1 (*CRB1*) gene is one of the human homologs of the Crumbs gene in *Drosophila*. Mapped to chromosome 1q31.3, *CRB1* mutations have been associated with a variety of retinal degenerations, including Leber congenital amaurosis (LCA), early-onset rod–cone dystrophy (EORCD), retinitis pigmentosa (RP), and cone–rod dystrophy (CRD) (1). Some atypical clinical features were also reported, such as isolated macular dystrophy and foveal retinoschisis (2–4), hyperopia and nanophthalmos (5, 6), optic disk drusen (6), and Coats-like vasculopathy (7). Thus, it is important to highlight those less common clinical features in order to improve both diagnosis and treatment.

In this paper, we report the case of a 7-year-old girl with biallelic heterozygous *CRB1* mutations, whose clinical features included macular retinoschisis, Coats-like retinal capillary leakage, short axial length, and angle-closure glaucoma (ACG). We also briefly review the current opinion on genotype and phenotype analyses as well as treatment approaches for *CRB1* mutation-related diseases. This case report was prepared following the CARE guidelines.

## Case Presentation

A 7-year-old girl with healthy, non-consanguineous parents was admitted to our hospital in 2016 with a complaint of poor visual acuity of both eyes, discovered by her mother occasionally. A history of premature birth, trauma, or systemic diseases was not reported by her parents. Her brother had normal vision.

**Figure 1:**
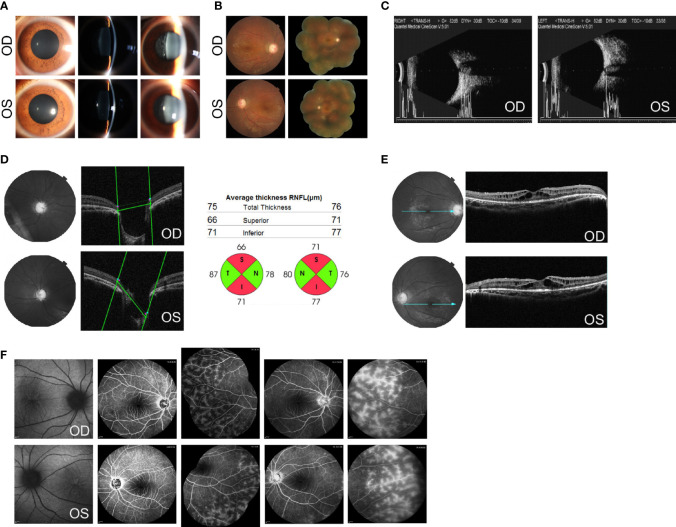
Ocular findings at the initial examination. **(A)** Slit-lamp aspects of the ocular anterior segment show moderate mydriasis, shallow anterior chamber, lucent lens, and vitreous opacities with cells in both eyes. **(B)** Fundus photographs show a C/D of 0.8 and dull macular reflex in the posterior pole, without pigmentary changes in both eyes. **(C)** B-mode ultrasound scanning shows a short axial length (20.0 mm for the right and 20.2 mm for the left) with optic disk excavation in both eyes. **(D)** Optical coherence tomography (OCT) shows an enlarged and depressed optic disk with RNFL thickness in both eyes. **(E)** OCT shows macular retinoschisis in the inner and outer nuclear layers, with the disruption of the ellipsoid zone and external limiting membrane within the fovea. **(F)** Fundus autofluorescence (AF) shows foveal hyperfluorescent specks in the right eye and streaks of radial hyper-AF originating from the central fovea in the left eye. The early and late phases of FA show spoke-wheel-like dye accumulation without leakage within the fovea and peripheral retinal telangiectasia with mild leakage in both eyes. OCT images of the optic disk and values of RNFL thickness were acquired using Topcon 3D OCT-2000 (Topcon Corporation, Tokyo, Japan). OCT images of the macula were acquired using Heidelberg SPECTRALIS OCT (Heidelberg Engineering, Heidelberg, Germany). AF images were acquired using Heidelberg SPECTRALIS HRA (Heidelberg Engineering, Heidelberg, Germany).

No abnormality was found by general physical examination. Her best-corrected visual acuity (BCVA) was 20/400 in the right eye and counting fingers (CF) at 30 cm in the left eye. The intraocular pressure (IOP) was 38 mmHg in the right eye and 35 mmHg in the left eye measured by Goldmann applanation tonometry. Slit-lamp examination showed mild conjunctival hyperemia. The pupil was around 4 mm in diameter, with delayed light reflex in both eyes. A shallow but clear anterior chamber was observed, combined with a lucent lens and vitreous opacities with cells (**Figure 1A**). Fundus evaluation showed a cup–disk ratio (C/D) of 0.8 and dull macular reflex in the posterior pole, with no pigmentary changes in the peripheral retina, in both eyes (**Figure 1B**). Gonioscopy further revealed closed anterior chamber angles in the nasal quadrant and narrow angles in other quadrants, with no signs of hyperpigmentation or neovascularization. The central cornea thickness was 606 μm in the right eye and 612 μm in the left.

Imageological examinations were followed to figure out the possible reasons for high IOP, enlarged C/D, and vitreous opacities. Her axial length, measured by a B-mode ultrasound scanner, was 20.0 mm for the right eye and 20.2 mm for the left eye, respectively, with optic disk excavation in both eyes (**Figure 1C**). The thickness of the retinal nerve fiber layer (RNFL), tested by optical coherence tomography (OCT), was significantly thinner on the superior and inferior side around the optic papilla bilaterally, compared with the normal value databank (**Figure 1D**). The macular region showed marked cystoid spaces in the inner and outer nuclear layers. Within the regions of the fovea, the disruption of the ellipsoid zone and external limiting membrane was also noted (**Figure 1E**). On fundus autofluorescence (AF), there were several hyperfluorescent specks within the fovea in the right eye and streaks of radial hyper-AF originating from the central fovea in the left eye (**Figure 1F**). Fluorescein angiography (FA) revealed no signs of optic disk or macular leakage apart from a little spoke-wheel-like dye accumulation within the fovea, as well as retinal telangiectasia with mild leakage of the peripheral retina in both eyes (**Figure 1F**).

The abnormal ocular phenotype included bilateral anterior angle closure, short axial length, optic atrophy, macular retinoschisis, and Coats-like response in the peripheral retina. In consideration of the later age of onset, normal size of the cornea without Haab’s striae, and closed anterior chamber angles, the diagnosis of primary congenital glaucoma were excluded. Further laboratory investigations referring to rheumatism, ankylosing spondylitis, tuberculosis, or other systemic autoimmune or infectious diseases were all negative, indicating no systemic abnormality. Therefore, the proposed diagnosis was childhood glaucoma associated with non-acquired ocular anomalies.

**Figure 2:**
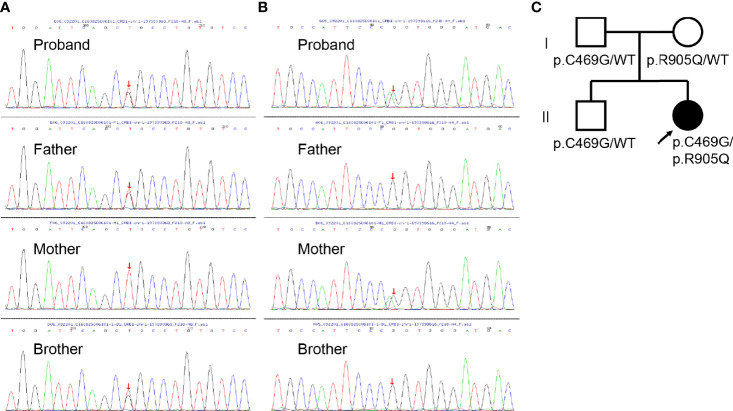
Genomic sequencing and pedigree of the present family. **(A)** Sanger sequencing revealing the heterozygous c.1405T>G mutation (red arrow) in *CRB1* detected in the proband, her father, and brother. **(B)** Sanger sequencing revealing the heterozygous c.2714G>A mutation (red arrow) in *CRB1* detected in the proband and her mother. **(C)** Pedigree of the present family with the proband indicated (black arrow). Squares indicate men, circles women, shaded symbols individuals with symptoms, and unshaded ones individuals without any symptoms.

To further investigate the pathology, DNA samples were screened to detect mutations in the most frequently involved genes in retinal diseases (**Supplementary Table S1**). The genetic testing showed combined heterozygous missense mutation (paternal p.C469G and maternal p.R905Q) in *CRB1* in the affected girl (**Figures 2A–C**), which might account for her clinical features including macular retinoschisis, short axial length, and Coats-like vasculopathy.

Initial treatment involved the following combined medications: beta-blockers, carbonic anhydrase inhibitors, alpha agonists, and finally prostaglandin analogs. Laser peripheral iridectomy (LPI) was performed to achieve a lower IOP fluctuation in both eyes. However, the IOP was beyond control with the maximum tolerated medications 1 year later, and the girl was treated with trabeculectomy combined with mitomycin C in both eyes one after another. In 2018, decreased bleb function was noted, and secondary surgery including phacoemulsification, intraocular lens (IOL) implantation, anterior vitrectomy, surgical peripheral iridotomy, and visco-goniosynechialysis was performed in both eyes. Then, routine follow-up based on the IOP and visual function was advised.

**Figure 3:**
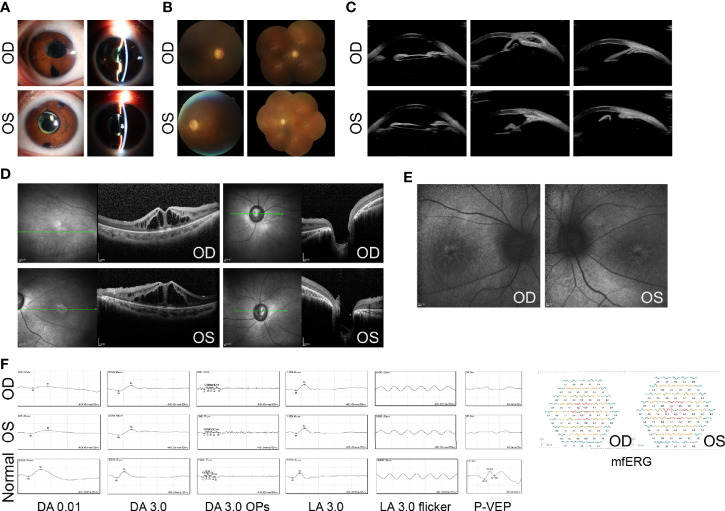
Ocular manifestations after treatment. **(A)** Slit-lamp examination shows limited functioning filtering bleb, deep anterior chamber, and IOLs in both eyes. **(B)** Fundus photographs show a pale optic disk with C/D of 0.9–1.0 in both eyes. **(C)** The UBM test shows closed anterior chamber angles in both eyes. **(D)** OCT shows more serious cystoid changes within the macula and optic disk excavation in both eyes. **(E)** AF reveals hyper-AF streaks and mass within the central fovea in both eyes. **(F)** ERGs show delayed and reduced waves for each recording, while PVEP potentials are undetectable in both eyes. mfERG shows decreased central amplitude density in both eyes. Age-matched normal ERGs and PVEP are shown for comparison. DA, dark-adapted; OPs, oscillatory potentials; LA, light-adapted. UBM images were acquired using Aviso (Quantel Medical, Cournon d’Auvergne, France). All OCT images were acquired using Heidelberg SPECTRALIS OCT (Heidelberg Engineering, Heidelberg, Germany).

In August 2020, the BCVA was 20/400 in both eyes, and the IOP was 18 mmHg in the right and 17 mmHg in the left (combined with 0.5% timolol BID and 1% brinzolamide TID). Slit-lamp examination revealed a limited functioning filtering bleb in both eyes. The anterior chamber was deep and IOL was in the capsular bag (**Figure 3A**). A pale optic disk was found, with a C/D of 0.9–1.0 (**Figure 3B**). An ultrasound biomicroscope (UBM) test further revealed closed anterior chamber angles in all quadrants in the right eye and in the nasal quadrant in the left eye (**Figure 3C**). OCT revealed more serious cystoid changes and disrupted structures of the outer retina within the macula, as well as excavation of the optic disk (**Figure 3D**). Blue AF indicated retinal pigment epithelium (RPE) atrophy in the macula (**Figure 3E**). For electrophysiological testing, the pattern visual evoked potential (PVEP) P100 component was undetectable, while delayed and reduced waves were found in each recording on full-field electroretinography (ERG) (**Figure 3F**). Decreased central amplitude density in multifocal ERG (mfERG) in both eyes was found as well (**Figure 3F**).

## Discussion

**Figure 4:**
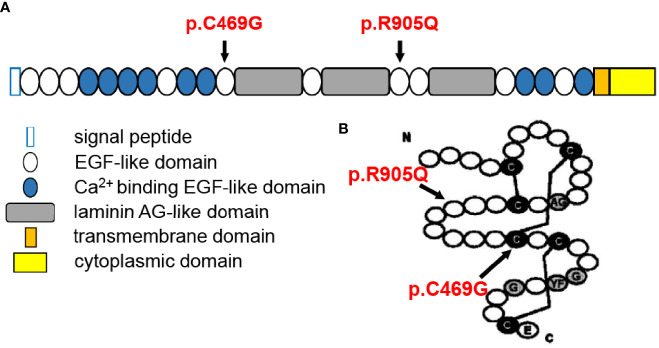
Structural illustration of canonical CRB1-A protein showing the location of the two mutations [diagrammatic drawings are modified and cited from den Hollander et al. (10) and Bujakowska et al. (11)]. **(A)** Position of the two mutations in relation to CRB1-A protein domains, showing that both mutations are located at the EGF-like domain. **(B)** Structure of the EGF-like domain with indications of the present mutations, showing that p.C469G mutation might disturb the formation of a disulfide bond between cysteine molecules.

The Crumbs protein was first identified in *Drosophila*. In mammals, the CRB family consists of four members, namely, *CRB1*, *CRB2*, *CRB3A*, and *CRB3B* (8). The prototype CRB protein is a single transmembrane protein with a large extracellular domain including a signal peptide sequence, 19 epidermal growth factor (EGF)-like domains, and 3 laminin A globular (AG)-like domains. The cytoplasmic domain includes conserved FERM- and PDZ-binding motifs (9) (**Figure 4A**). They are localized primarily at the subapical region above the adherens junctions (AJ) between photoreceptors, Müller glia cells, and between photoreceptors and Müller glial cells (12) and form the core of the CRB complex which maintains the correct apical–basal polarity and adhesion of retinal neuro-epithelium (8). Therefore, any possible disruption of *CRB1* might break the AJ structure and cell polarity, induce photoreceptor injury and finally lead to retinal degeneration resulting in vision impairment.

To date, 553 variants in *CRB1* have been described [based on the Leiden Open Variation Database (LOVD), https://databases.lovd.nl/shared/genes/CRB1, updated April 13, 2022], of which 450 were labeled pathogenic/pathogenic-like, resulting in a range of retinal disease phenotypes. Ehrenberg et al. (1) reviewed that *CRB1* mutation was detected in around 0%–17% of LCA patients, 7% of EORCD patients, and 0%–6.5% of RP patients. CRB1-associated RP, designated “RP12”, appears severe, autosomal recessive and is characterized by a preserved para-arteriolar retinal pigment epithelium (PPRPE) in the early-to-middle stages of disease (13). For LCA, *CRB1* mutations cause LCA8, characterized by the following retinal hallmarks: nummular pigment clumps, yellow-white retinal dots, and salt-and-pepper retinal pigmentation (1, 14). Notably, besides those typical phenotypes, a series of rare consequences, including isolated maculopathy, keratoconus, hyperopia or nanophthalmos, and Coats-like vasculopathy, have recently been observed. Firstly, based on previous reports, *CRB1*-associated maculopathy is characterized by the presence of intraretinal cysts in the inner and outer retinal layers, which could be detected as spoke wheel appearance at the fovea with marked schitic changes at OCT (4, 15–21). In some cases, these changes were described as “macular edema” (18). However, as what was shown in our case, there might be no macular leakage on FFA. Khan et al. (17) described that intraretinal cysts tended to appear in younger patients than older ones, as the latter presented outer retinal degeneration and macular atrophy instead. Mucciolo et al. (20) reported a long-term follow-up of a patient with *CRB1*-associated maculopathy and found a spontaneous passage from a foveal schitic shape to a cystic profile and finally atrophic maculopathy, without signs of leakage on FFA, which might be regarded as the natural evolution in the course of the disease. Secondly, relative short axial length was another atypical appearance reported due to *CRB1* mutation. RP12 and LCA8 have been frequently associated with hyperopia (1, 5, 7, 21). Nanophthalmos has also been reported in *CRB1* mutants (6, 22). In a retrospective cohort study, 7 out of 55 patients with *CRB1* mutations had associated glaucoma (2). More recently, Liu et al. (23) and Abe et al. (24) have described cases with *CRB1*-associated RP combined with primary ACG. Therefore, it is indicated that *CRB1* mutation may account for the girl’s short axial length, crowded anterior segment, and ACG. Another point in our case is retinal peripheral telangiectasia, which might refer to an early stage of Coats-like response or inflammatory features. On the one hand, it is reported that *CRB1* plays a role in neurovascular interaction that regulates layered vascular network development and integrity (25). den Hollander et al. (7) suggested that *CRB1* mutations were strongly associated with the development of Coats-like exudative vasculopathy in patients with RP. On the other hand, the vasculopathy observed in *CRB1* mutations could also be affected by microenvironmental factors. Murro et al. (26) reported a case of *CRB1*-associated retinal dystrophy characterized by vitritis, retinal capillaritis, and cystoid change of macular. Bujakowska et al. (9) analyzed published phenotypes of *CRB1* mutants and showed that the presence/absence of Coats-like vasculopathy did not reveal a particular mutation pattern, which suggests the involvement of additional genetic and/or environmental modifying factors. Notably, similar signatures, including retinoschisis, mild peripheral angiographic leakage, and ACG, could also be found in autosomal recessive bestrophinopathy (ARB) caused by mutations in both alleles of the *BEST1* gene (27, 28). Therefore, genetic testing is essential for differential diagnosis.

Though scientists attempted to understand the phenotype–genotype correlation of *CRB1* mutations, current meta-analyses failed to conclude any clear correlation, apart from the agreement that null mutations were more likely to cause more severe phenotypes, indicating additional modifying factors (1, 2, 9, 10, 16, 17). Moreover, it is reasonable to consider that mutations that occurred in key locations relating to protein special structure are more dangerous. Recently, it has been found that there are three main CRB1 isoforms in the human retina: the canonical CRB1-A localized in Müller glial cells (12 exons), the potentially secreted CRB1-C, and a new isoform CRB1-B predominant in photoreceptors (7 exons) (29). Therefore, the positions of mutation may also affect distinct isoforms and lead to different phenotypes. Indeed, Mairot et al. (30) reported that the phenotype in CRB1 patients might be dependent on the severity of Müller cell impairment related to isoform diversity. In addition, experiments based on gene knockout mouse models indicated a modifying role of CRB2 (31–34). In our case, each of the two mutations was inherited paternally and maternally, respectively, and both were located in EGF-like domains (**Figure 4A**). These two mutations have been previously reported to be associated with LCA (11, 35). The p.C469G mutation affects exon 6 of CRB1-A and, therefore, affects all three isoforms and might disturb the formation of a disulfide bond between cysteine molecules leading to structural alteration. The p.R905Q affects exon 8 of CRB1-A and, therefore, only affects CRB1-A and CRB1-B but does not affect CRB1-C(**Figure 4B**).

The key points in the management of this case are to stabilize the IOP, monitor macular atrophy, and prevent peripheral capillary leakage. It is still controversial whether the non-edema macular cysts in retinal dystrophy need to be treated or not. Some studies showed that oral uptake of carbonic anhydrase inhibitors (i.e., acetazolamide) was effective in promoting resolution, as they could regulate fluid movement from the retina across the RPE to the choroid and strengthen retinal adhesiveness (4, 36, 37). However, studies also revealed a spontaneous resolution of the cystic abnormalities toward atrophic evolution, which indicated the minimal necessity for drug treatment (20). In this context, long-term follow-up is required to monitor disease progression and adjust therapeutic strategies. Recent studies on mouse models take new insights into gene therapy for *CRB1* using adeno-associated viral (AAV) vector-based gene augmentation (38–41). Moreover, the use of CRISPR/Cas9 to correct specific point mutations in patients is also discussed as a potentially viable method (12). In addition, *CRB1* patient-induced pluripotent stem cell (iPSC)-derived human retinal organoids could mimic the retinal phenotype and might serve as good models for testing gene therapeutic approaches including both gene augmentation and gene editing (42). Recently, a French biotech company called Horama has signed an exclusive license agreement with Leiden University Medical Center targeting *CRB1* gene mutations to treat inherited retinal dystrophies, which is expected to initiate a phase I/II clinical study with the drug candidate in 2023 (41). Those efforts may bring new approaches to a better outcome for patients suffering from *CRB1* mutation.

## Conclusion

In this paper, we report a rare phenotype of biallelic heterozygous *CRB1* mutations which are characterized by macular retinoschisis, Coats-like vasculopathy, short axial length, and ACG. This case emphasizes the importance of securing a genetic diagnosis for patients with combined manifestation in anterior and posterior segments, especially when it appears as cryptogenic retinal atrophy, schisis, or cystic profile. Moreover, it highlights the vital role of *CRB1* in maintaining normal macular structure and eyeball development.

## Data Availability Statement

The datasets presented in this article are not readily available because of ethical and privacy restrictions. Requests to access the datasets should be directed to the corresponding author.

## Ethics Statement

Written informed consent was obtained from the individual(s), and minor(s)’ legal guardian/next of kin, for the publication of any potentially identifiable images or data included in this article.

## Author Contributions

J-XS, DH, JZ, Y-SW, JW, X-JS, and XH examined and treated the patient. H-XY and X-JS helped with the genome analysis. J-XS and H-XY drafted the manuscript. XH initiated the concept, supervised the writing of the manuscript, and was responsible for the clinical data. All authors contributed to the writing of the final manuscript.

## Funding

This study was supported by the National Natural Science Foundation of China (81900870 and 81371034).

## Conflict of Interest

The authors declare that the research was conducted in the absence of any commercial or financial relationships that could be construed as a potential conflict of interest.

## Publisher’s Note

All claims expressed in this article are solely those of the authors and do not necessarily represent those of their affiliated organizations, or those of the publisher, the editors and the reviewers. Any product that may be evaluated in this article, or claim that may be made by its manufacturer, is not guaranteed or endorsed by the publisher.
