# Supplementary material for: Biallelic Heterozygous Mutations in Crumbs Homolog-1 Gene Associated With Macular Retinoschisis and Angle-Closure Glaucoma: A Case Report and Literature Review
Source: Front Ophthalmol (Lausanne). 2022 Jun 3;2:902898. doi: 10.3389/fopht.2022.902898 (PMC11182100; doi:10.3389/fopht.2022.902898)
Supplement: Supplementary file 1 [file Table_1.docx]

Supplementary Information

Table S1 List of analyzed genes

| MT-ND1 | G3460A | MT-ND1 | C4171A | MT-ND6 | C14482G | MT-TI | G4308A |
| --- | --- | --- | --- | --- | --- | --- | --- |
| MT-ND1 | G3635A | MT-ND4L | T10663C | MT-ND6 | T14484C | MT-TL1 | A3243G |
| MT-ND1 | G3700A | MT-ND4 | G117788A | MT-ND6 | A14495G | MT-TL2 | G12315A |
| MT-ND1 | G3733A | MT-ND6 | C14482A | MT-ND6 | C14568T | MT-TN | G5703A |
| ABCA4 | CACNA1F | FLVCR1 | KIF7 | OPA6 | PRPH2 | SDCCAG8 | TRPM1 |
| ABCB6 | CACNA2D4 | FSCN2 | KLHL7 | OPN1LW | RAB28 | SEMA4A | TSPAN12 |
| ABHD12 | CC2D2A | FZD4 | LCA5 | OPN1MW | RAX2 | SHH | TTC8 |
| ADAM9 | CCDC28B | GDF3 | LRAT | PAX6 | RBP3 | SLC24A1 | TTLL5 |
| AHI1 | CDH23 | GDF6 | LRIT3 | PCDH15 | RD3 | SLC25A4 | TUBA8 |
| AIPL1 | CDHR1 | GNAT1 | LRP5 | PCYT1A | RDH12 | SLC38A8 | TULP1 |
| ARL13B | CEP290 | GP1BA | LZTFL1 | PDE6A | RDH5 | SLC7A14 | UCHL1 |
| ARL2BP | CEP41 | GPR179 | MAK | PDE6B | RGR | SNRNP200 | UNC119 |
| ARL6 | CERKL | GPR98 | MERTK | PDE6C | RHO | SOX2 | USH1C |
| BBIP1 | CIB2 | GRK1 | MKKS | PDE6D | RIMS1 | SPATA7 | USH1G |
| BBS1 | CISD2 | GRM6 | MKS1 | PDE6G | RLBP1 | STRA6 | USH2A |
| BBS10 | CLRN1 | GUCA1A | MYO7A | PDE6H | ROM1 | TBX1 | VSX2 |
| BBS12 | CNGA1 | GUCA1B | NBAS | PDZD7 | RP1 | TCTN1 | WDPCP |
| BBS2 | CNGB1 | GUCY2D | NDP | PITPNM3 | RP2 | TCTN3 | WFS1 |
| BBS4 | CNNM4 | HARS | NEK2 | PLK1S1 | RP9 | TENM3 | YAP1 |
| BBS5 | CRB1 | IDH3B | NMNAT1 | POC1B | RPE65 | TIMM8A | ZNF408 |
| BBS7 | CRX | IFT27 | NPHP1 | POLG | RPGR | TIMP3 | ZNF423 |
| BBS9 | CSPP1 | IKBKG | NPHP4 | POLG2 | RPGRIP1 | TMEM126A | ZNF513 |
| BEST1 | CYP4V2 | IMPDH1 | NR2E3 | PRCD | RPGRIP1L | TMEM138 |  |
| C10orf2 | DFNB31 | IMPG2 | NR2F1 | PROM1 | RQCD1 | TMEM216 |  |
| C2orf71 | DHDDS | INPP5E | NRL | PRPF3 | RRM2B | TMEM231 |  |
| C5orf42 | DNA2 | IQCB1 | NYX | PRPF31 | RS1 | TMEM237 |  |
| C8orf37 | ELOVL4 | KCNJ13 | OFD1 | PRPF4 | RYR1 | TMEM67 |  |
| CA4 | EYS | KCNV2 | OPA1 | PRPF6 | SAG | TOPORS |  |
| CABP4 | FAM161A | KIF11 | OPA3 | PRPF8 | SALL2 | TRIM32 |  |
